# A study of the user's perception of economic value in nursing visits to primary care by the method of contingent valuation

**DOI:** 10.1186/1471-2296-12-109

**Published:** 2011-10-03

**Authors:** Jesús Martín-Fernández, Francisco Javier Pérez-Rivas, Tomás Gómez-Gascón, Isabel del Cura-González, Eugenia Tello Bernabé, Gemma Rodríguez-Martínez, Elena Polentinos-Castro, Julia Domínguez-Bidagor, Gloria Ariza-Cardiel, Juan Francisco Conde-López, Milagros Beamud-Lagos, Óscar Aguado-Arroyo, Teresa Sanz-Bayona, Ana Isabel Gil-Lacruz

**Affiliations:** 1 Family Medicine Unit, West Area, Primary Care, Madrid Health Service, Spain; 2 Quality Unit, Primary Care, Madrid Health Service, Spain; 3 Guayaba Health Centre, Primary Care, Madrid Health Service, Spain; 4 Research Unit, Primary Care, Madrid Health Service, Spain; 5 El Naranjo Health Centre, Primary Care, Madrid Health Service, Spain; 6 Condes de Barcelona Health Centre, Primary Care, Madrid Health Service, Spain; 7 Family Medicine Unit, North Area, Primary Care, Madrid Health Service, Spain; 8 Prevention and Promotion Service, Madrid Health Service, Spain; 9 Family Medicine Unit, West Area, Primary Care, Madrid Health Service, Spain; 10 San Martín de Valdeiglesias Health Centre, Primary Care, Madrid Health Service, Spain; 11 El Greco Health Centre, Primary Care, Madrid Health Service, Spain; 12 Francia Health Centre, Primary Care, Madrid Health Service, Spain; 13 Management Unit, West Area, Primary Care, Madrid Health Service, Spain; 14 Economics Assistant Professor, Zaragoza University, Zaragoza, Spain

## Abstract

**Background:**

The identification of the attribution of economic value that users of a health system assign to a health service could be useful in planning these services. The method of contingent valuation can provide information about the user's perception of value in monetary terms, and therefore comparable between services of a very different nature. This study attempts to extract the economic value that the subject, user of primary care nursing services in a public health system, attributes to this service by the method of contingent valuation, based on the perspectives of Willingness to Pay (WTP) and Willingness to Accept [Compensation] (WTA).

**Methods/Design:**

This is an economic study with a transversal design. The contingent valuation method will be used to estimate the user's willingness to pay (WTP) for the care received from the primary care nurse and the willingness to accept [compensation] (WTA), were this service eliminated. A survey that meets the requisites of the contingent valuation method will be constructed and pilot-tested. Subsequently, 600 interviews will be performed with subjects chosen by systematic randomized sampling from among those who visit nursing at twenty health centers with different socioeconomic characteristics in the Community of Madrid. The characteristics of the subject and of the care received that can explain the variations in WTP, WTA and in the WTP/WTA ratio expressed will be studied. A theoretical validation of contingent valuation will be performed constructing two explanatory multivariate mixed models in which the dependent variable will be WTP, and the WTP/WTA relationship, respectively.

**Discussion:**

The identification of the attribution of economic value to a health service that does not have a direct price at the time of use, such as a visit to primary care nursing, and the definition of a profile of "loss aversion" in reference to the service evaluated, can be relevant elements in planning, enabling incorporating patient preferences to health policy decision-making.

## Background

The definition of health policies has an ethical dimension that is not faithfully reflected in the rest of planning, and which is probably provided by the conception of health as a fundamental good for human beings [1]. The main challenge of these policies is to approach the health needs of the population as effectively and efficiently as possible. A key element in the definition of health policies is the availability of resources and their distribution among the different services that provide them.

In our setting, the health system if organized as a national system and provides its services through an organization at two levels, so-called Specialized Care (SP), and Primary Care (PC). Although for nearly 30 years primary care has been defined as the entry-door structure to the system and the management of the health needs of patients, in recent years this care level has undergone a significant process of "disinvestment" [2]. Valuation of the public health system, its needs, its effectiveness and efficiency and its role in the social structure is a highly complicated task. But this tendency to reduce investment in primary care is not justified by an analysis of the social needs expressed by health demand, because demand for primary care is high, and increasingly so among those with a poorer perception of their state of health [3]. Nor is it supported by the study of people's preferences, or by the results of the impact on health at each care level in the population as a whole, because as far as it is known, investment in primary care is accompanied by more efficient health results [4].

In the context of reducing investment, a study of the perception of value perceived by users of primary care services appears timely. There is no way to determine this perception of value when the service evaluated is not provided under market conditions and cannot be identified by a price tag. This is why the contingent valuation method is used to answer these questions. The contingent valuation method attempts to simulate a hypothetical market by surveying consumers. The objective of the interview is to create a hypothetical scenario in which the subjects interviewed represent demand and the interviewer plays the role of supply. The contingent valuation method is based on theoretical economics and assumes that individual preferences can be interpreted in the form of a function of utility, where two different states (initial and final) can be compared in terms of the changes in the utility function. Willingness to Pay (WTP) and Willingness to Accept [Compensation] (WTA) are the measures proposed in the framework of welfare theory, which attempt to value, in monetary terms, gains and losses in utility an individual experiences when a project or intervention is introduced or eliminated. Initially, this method was utilized to value public goods not subject to the market, but today its use has been extended to value quasi-public goods, such as those provided by health services. The contingent valuation method is especially suitable in the case of the valuation of health care because it includes use values and non-use values, that is, the value derived from the consideration of the product as a consumer good and those related to the existence of the service itself [5]. The contingent valuation method has been employed in the field of primary care to estimate the willingness to a defray improvements in health care in systems under development [6], to evaluate health promotion programs [7], mental health care [8], the extension of certain health coverage [9], and the willingness to pay for informal care [10]

The perception of value of the service can be studied as a whole or in its components. The Basic Care Unit (BCU), also called the Family Care Unit (FCU), constitutes the basic care pillar of primary care, and is composed of a family physician and a family or communitary nurse. In a previous study we evaluated the perception of economic value the user expressed of the visit to the family physician. This made clear that not setting a direct payment at the moment use of the service, that is, the absence of a price tag, did not represent the absence of the perception of value [11]. But no information is available about the perception of value the user has of primary care nursing services. Knowing the monetary value that users of a health system assign to a certain service could be useful in deciding the level of investment and "amount of need" to which a response must be given, this constituting an unmistakable expression of the user's own preferences.

The value attributed to a good or service by contingent valuation methodology can be studied from the perspective of willingness to pay (WTP) for the enjoyment of the good, or by willingness to accept [compensation] (WTA) for its removal. Being valuations of the same good, the two approaches should produce similar values. However, we know that values obtained by WTA are consistently higher than those expressed by WTP, when valuing the same good [12,13].

There are several theories to explain these differences between WTP and WTA. Attempts have been made to explain this by economic theory; this is because certain goods cannot be easily substituted (the utility they provide cannot be easily replaced by another consumption), which requires greater compensation for its loss [14]. But perhaps the most studied idea is "loss aversion." An experimental situation has been described, known as the "endowment effect," according to which, subjects who have been provided with a good and are asked how much money they would have to be paid to give it up, ask for amounts that far exceed the amounts offered by similar subjects who have not had the opportunity to buy the good. This theory emphasizes resistance to loss as an explanation of the difference between WTP and WTA. Loss aversion can be understood assuming that the marginal utility of the individual decreases. That is, an improvement in health produces a change in utility lower in absolute value, a lower marginal utility, than a loss of health in the same degree [15]. This leads us to think that, knowing the differences between WTP and WTA for a good or service, can help us understand not only the value, but also the possible resistance to "loosing" a good or service. This is why the valuation of certain health services from the perspectives of WTP and WTA can be a useful tool in health planning.

In summary, we find ourselves in a setting of providing public health services, the user of which is not used to valuating in terms that enable comparing them with other goods or services. The planner puts a price on this good by references, some objective (health results), others subjective (satisfaction), useful, but not valid when making these kinds of comparisons. Contingent valuation methodology can provide us with information about the user's perception of value in monetary terms and, therefore, comparable between services of a very different nature. Additionally, this methodology enables us to evaluate to a certain degree adherence to the service by evaluation of "resistance to loss", specifically, in the evaluation of the service provided by the nurse in primary care. Knowing these two circumstances, perception of economic value and resistance to loss, would be most valuable in planning health provisions and interpreting the preferences of the people to which this service is addressed.

## Methods/Design

### Design

This is an economic study with a transversal design. The contingent valuation method will be used to estimate willingness to pay (WTP)/willingness to accept [compensation] (WTA), for health care received in a primary care nursing visit in a public health system in the Community of Madrid. This is done by personal interviews of the users of these services.

### Subjects of study

Included are persons older than 18 years of age who use the primary care nursing service. To be included they must have experience in the exchange of goods in market conditions, to be able to understand the scenarios posed, understand Spanish correctly, and consent to be interviewed.

Criteria for exclusion are not understanding the language, being unable to give their consent or not understanding the objective of the interview.

### Sample size and obtaining the sample

If we wish to estimate the parameter (WTP or WTA) with sufficient precision so that the confidence interval will have an amplitude of less than 15% of the standard deviation (30% precision), for a confidence of 95%, and with the common formulas for this calculation, we will need to include 170 subjects. However, we will be studying groups of subjects (health centers) in which the variability of the parameter being studied will be less among the subjects of the group and greater among the subjects of the different groups. This is the definition of the "design effect," which causes estimates to lose efficiency compared with purely random sample selection systems. Measuring the size of the design effect depends on the relationship between the intragroup and intergroup variances, specifically what is called the Intraclass Correlation Coefficient (ICC), and the number of subjects studied in each group. In a previous paper that evaluated the WTP for a medical visit, the value of the ICC was 0.048. Estimating an unfavorable scenario in which the ICC was 50% greater (ICC approx. 0.075), and including an approximate number of 30 patients/center, the design effect would be equal to 3.18, which would bring us to include 543 subjects (170 × 3.18). Since there has to be a percentage of incomplete interviews, or patients who fail to reveal the necessary information for subsequent analyses, and given that the marginal cost of each new interview is small, and is not an inconvenience for the subject interviewed, our inclusion objective will be 600 patients. This represents the recruitment of about thirty patients at twenty health centers.

#### Sampling

The study sample will be selected by systematic randomized sampling carried out at health center nursing visits, after first requesting their consent to participate. The health centers are stratified based on two characteristics, rural or urban populations and average income of the district in which they are located. Health centers will be selected from the upper income tercile and lower income tercile, based on data published in the last report available from the National Statistics Institute for the Community of Madrid. This will ensure the inclusion of patients from health centers equally distributed from the two ends of the income distribution terciles, and at least one in five with rural characteristics.

At each health center, on the date of the study, a number of patients will be selected by systematic randomized sampling from an anonymous randomized list of patients with nursing visit appointments, until the number fixed for that is center is reached.

### Measuring tools and variables included

The contingent valuation method requires individual interviews to estimate WTP and WTA. The interview will be performed by skilled personnel, trained in the methodology of the study and who are from outside the health system. The place of the interview will be the health center itself, but outside the care area (library, meeting room, etc.). In case of house calls, patients will be previously contacted by their nurse who will obtain their informed written consent, and the interview will be over the telephone.

A survey that can be filled out in approximately twenty minutes will be handed out at the interview.

First of all, the adaptation and pilot test of the survey will be performed. The quality of the information collected depends in large measure on having an information collecting tool that will clarify for the persons studied the following aspects: objective of the study, product attributes, context of the exchange to be valued, range and certainty of payment and social context. The survey will have a first section that will explain the objective of the study as the economic value of the nursing visit, and certain attributes will be defined such as the universality of care, accessibility and, broadly speaking, the services portfolio. The second section will be the economic valuation itself. Two scenarios will be presented here: the first makes explicit the WTP for the service in question. The second will ask about WTA, if this service were to be eliminated. The monetary measure of the variation in the utility communicated will refer to a compensatory variation framework (compensation required so that, following the exchange, the utility remains) of the classification proposed by O'Brien and Gafni [16]. The payment format will be a direct payment. The type of question will be in a rank format or "payment card," as this format causes the user to behave as if he or she would be in a setting in which the same product was being sold at different prices [17]. The questions will be posed in two parts, the first presenting only three options that include the terciles of the values obtained in the pilot test, and a second payment card that reduces the response given to at least five values equidistant for each tercile; this limits any possible bias for inducing a response.

The cutoff points will be decided by consensus of the research team following the pilot test. The questions in the pilot test on WTP and WTA will be made in an open format. In the questionnaire, the payment cards will include the value 0, and subsequently there will be a question that differentiates so-called "protest zeros" (do not accept the question itself), from the inability to make the payment.

The third and final section of the survey will collect, at least, the following characteristics, which will be transformed into independent variables in the study:

- Characteristics of the center: Rural/urban; Average income of the area

- Sociodemographic characteristics:

- Age in years.

- Sex: male/female

- Employment situation: active worker/unemployed or student/pensioner not retired/retired

- Education level attained: no education/primary/secondary/university

- Social class: (Categories I, from highly specialized professions to V, least specialized).

- Income level: sum of total weighted family income (Total/N^0.5^)

- Characteristics of use of service:

- Frequency: Number of nursing visits in last year

- Type of visit (depending on place of care): health center/domicile

- Type of visit (depending on type of service): promotion and prevention activities; care/monitoring pathologies/chronic processes; identification/monitoring of care plans; diagnostics and therapeutics procedures.

- Need of use of the service:

- Number of chronic pathologies suffered (requiring care for more than six months)

- Hospital admissions in last year (yes/no)

- Health-Related Quality of Life (HRQL) measured by EuroQol 5D

- Type of living arrangements: alone/with partner only/family nucleus/extended family nucleus. Family APGAR

- Caregiver of another person due to illness or incapacity. Yes/no

- Satisfaction with service: AMABLE questionnaire

### Analysis of the data

The value provided by WTP and WTA will be selected for the descriptive analysis and these will be treated as continuous variables, which will be defined by their measures of central tendency and dispersion.

Continuous independent variables will be defined by the same parameters, and discrete variables by an estimate of the proportions and their confidence intervals of 95%. For an estimate of the confidence intervals the variance inflation factor caused by the type of sampling in conglomerates will be taken into account. Consistency of WTP and WTA will be evaluated in a subgroup of subjects by the intraclass correlation coefficient (ICC). To calculate the consistency of the response, the WTP and WTA question will be repeated by telephone to 100 randomly selected subjects, which enables estimating an ICC of around 0.9 with a minimum precision of 7.5%, with a confidence of 95% [18]. Evaluation of the consistency of the response, though this will depend considerably on interpersonal variability, will serve to evaluate the internal validity of the result [19]. The relationship between WTP and WTA will be measured by Pearson's correlation coefficient or Spearman's rho, as well as the ICC.

Univariate analysis will be performed to study the association of the independent variables with WTP, employing parametric tests for continuous variables that are distributed according to a normal distribution and non-parametric tests for the rest.

To validate the model and study the WTP/WTA relationship, two multilevel mixed linear models will be constructed. The variables referring to the individual will constitute the first level, and contextual variables (referring to the clusters or health centers) will make up the second level [20]. The model will be explanatory, given the design of the study. In case the contextual variables do not explain a significant part of the variance we will resort to classical multilevel models with robust estimators of the regression coefficients (Eicker-White estimator) that minimize the problems of heteroscedasticity [21]. The dependent variable of the models will be WTP or the WTP/WTA ratio, net or softened in its logarithmic expression. The independent variables will be introduced depending on their explanatory role in the model, and the final model will be chosen by the principles of maximum parsimony.

### Ethical and legal aspects

All the research process will be governed by the ethical principles contained in the Declaration of Helsinki (revision Seoul 2008). Those persons who are included will be asked for their consent in writing to participate in the study and will be advised that all the data will be stored and treated anonymously, complying with the requisites established in national legislation. The study has the favorable report of the Ethics Review Board of the Hospital Fundación Alcorcón.

## Discussion

Identifying the attribution of economic value to a health service that does not have a direct price at the time of use is relevant because it can be a way of incorporating patient preferences to health policy decision-making. It has been pointed out that one of the challenges of economic evaluation is the evolution from an expression of mere rationalism in the form of establishing normative behaviours to constituting a way of making preferences explicit [22], and it is in this framework that this project is designed.

Successfully identifying the value attributed to the service can only be complete validating the results in the construct of welfare theory [18], relating the values obtained to health needs, satisfaction with the service received and socioeconomic situation.

Defining a profile of "loss aversion" referring to the service evaluated is also relevant. Characterizing subjects who show less willingness to do without the service provides planners with another element of judgment of people's preferences. This approach has been used in our setting for the evaluation of the visit to the family physician with results that speak of the strength of the method [23].

As to potential limitations of the study we note questions of how representative the sample is, any information biases and those that pertain to the method itself.

With respect to how representative it is, because this is a sampling of users the probability of including those who most use the service increases and this can add more weight to use value in detriment of non-use values (altruistic and of opportunity or option).

Possible information biases could limit the results of the study to some extent. The collection of variables such as frequency or number of pathologies will be provided by the family nurse with the patient's consent, but expressing social and economic self-positioning may be problematic. To minimize this resistance, we will also use the card format, allowing the person interviewed to only have to answer the letter of the category in which that person's answer if included. The quality of the design of the questionnaire will also be determining. Because the description of the scenario can have implications for the WTP expressed [24], we have chosen as realistic a description as possible, referring to a good obtained at the previous moment. If we can make a perfect description of the product, the market and the context of the transaction this will improve the validity and reliability of the result. This is why the design of the survey is viewed as one more phase of the research project.

Collecting WTP and WTA in a payment card format can produce biases, by varying in a specific range (yeah-saying bias). To limit this, the question is posed in two phases, each with a different payment card. The idealness and the advantages and disadvantages of the payment card compared with other formats and other methods to estimate WTP have been widely discussed; however, it is a commonly accepted tool [25]. Strategic bias and hypothetical bias, inherent to the contingent valuation method, cannot be avoided in this study. The first refers to the subject's tendency to express an unreal WTP, influenced by that person's intuition with respect to the study's objective (for example, refers to a low WTP if the person believes that this will influence the final price, if the service were ever paid for). We can value this tendency, but not avoid it, by studying how the WTP/WTA ratio behaves. The hypothetical bias reflects the difference in a subject's behavior when faced with hypothetical payments and in the real market. This bias is very difficult to establish, although it is more frequent in *ex-ante *studies than when the product being valued is known, which is our case. To evaluate the validity of the measure we will utilize the only approach feasible in this case. We will examine the congruence of the characteristics of the subject who expresses a certain WTP in the framework of economic welfare theory, and the reliability of the measure in a subgroup of patients.

Therefore, successfully identifying in this project the attribution of economic value to a health service, such as a visit to nursing in primary care, and the definition of a "loss aversion" profile referring to the service evaluated, can constitute, despite the shortcomings of this kind of design, elements of interest for planning health services in our setting, enabling decision-making that incorporates the preferences expressed by the system's users.

## Competing interests

None of the authors has any economic conflict of interest. All the authors, except AIGL, work in the public health system provider of the services being evaluated.

## Authors' contributions

JMF conceived and participated in the design of the study, draft the manuscript, and reviewed the final manuscript. FJPR, TGG, MICG, GRM, EPC, JDB, GAC, JFCL, and MBL participated in the design of the study and reviewed the final manuscript.

METB, OAA, MTSB, and AIGL contributed to the design of the study and reviewed the final manuscript.

## Pre-publication history

The pre-publication history for this paper can be accessed here:

http://www.biomedcentral.com/1471-2296/12/109/prepub
